# Reduction of the harmful NO_x_ pollutants emitted from the ship engines using high-pressure selective catalytic reduction system

**DOI:** 10.1007/s11356-024-33439-y

**Published:** 2024-04-26

**Authors:** Kubilay Bayramoğlu, Semih Yılmaz, Mustafa Nuran

**Affiliations:** 1 Department of Marine Engineering, Maritime Faculty, Zonguldak Bülent Ecevit University, Kdz. Ereğli, Zonguldak, 67300 Türkiye; 2 Department of Marine Engineering, Maritime Faculty, Dokuz Eylul University, Tınaztepe Campus, Buca, Izmir, 35390 Türkiye

**Keywords:** Marine transportation, HP-SCR, NO_X_ emissions, Pollutant reduction, Marine engine, CFD

## Abstract

Various techniques are used to reduce harmful pollutants such as NO_X_ emissions from ships. Selective catalyst reduction (SCR) systems are the most effective technique used to reduce NO_X_ emissions. In this study, the effects of an SCR reactor on NO_X_ emissions and performance in high-pressure selective catalytic reduction (HP-SCR) systems were investigated numerically. In numerical studies, the effects of SCR system diameter, output form, catalyst activation energy, mixing zone length, and location were investigated as parametric, and the most suitable system geometry was determined. The effects of geometric parameters and catalyst type on emission and performance such as NO_X_ reduction, NH_3_ slip, velocity, and pressure loss were investigated. It was determined that with increasing system diameter, whereas the NO_X_ reduction performance increased depending on exhaust velocity, the pressure drop decreased, and the most suitable system diameter was determined as 780 mm. Furthermore, the obtained results showed that the performance of NO_X_ reduction decreased after 2 × 10^6^ kJ/kmol activation energy, and the most suitable SCR output form was conical geometry. In terms of the environment, this study will contribute to achieving the UN Sustainable Development Goals such as climate action (SDG 13).

## Introduction

Approximately 90% of the foreign trade volume in the world is transported by sea on a scheduled basis. A large amount of dry cargo, liquid and gas, and containerized materials are transported by sea, where commercial ships are mostly used. The fuel consumption of ships, which have a large share in trade, and their role in air pollution due to this fuel consumption are also high (Deng et al. 2021). IMO has introduced various regulations on the environmental effects and reduction of greenhouse gases originating from ships (Smith et al. 2015). The Tier III regulation, which was put into effect on January 1, 2016, within the scope of IMO MARPOL Annex VI, has aimed to reduce NO_X_ emissions from ships by 76% compared to Tier II (IMO 2022). There are many techniques such as changing engine combustion parameters, using alternative fuels, and integrating after-treatment systems to reduce NO_X_ emissions from ship diesel engines (Gong et al. 2017; Bayramoğlu et al. 2019; Lu et al. 2020; Bayramoğlu and Yılmaz 2021; Öztürk and Can 2022). SCR systems are one of the most effective techniques that reduce NO_X_ emissions by 90%.

The SCR system is an after-treatment system that reduces NO_X_ emissions from the exhaust by injecting the urea-water solution (UWS) into the exhaust gas. First, the water in the UWS evaporates with the temperature of the exhaust gas and then undergoes thermolysis and hydrolysis reactions forming NH_3_. The NH_3_ formed reacts with NO_X_ emissions in the catalyst to form free N_2_ and H_2_O (Birkhold et al. 2007; Lee 2018). However, to provide NH_3_ conversion in SCR systems, the system design and UWS injection must be well determined. While system design parameters affect the efficiency of hydrolysis and thermolysis reactions, UWS influences the amount of NH_3_ formed. An insufficient amount of NH_3_ causes less NO_X_ reduction performance, and excess NH_3_ causes NH_3_ slip in the exhaust (Choi et al. 2015; Sung et al. 2020). In the literature, various parametric studies have been conducted to determine the most suitable NH_3_ conversion and UWS injection parameters in SCR systems.

The most common technique that provides efficient NH_3_ conversion in SCR systems and is widely used is the use of a static mixer. The static mixer provides effective mixing of the NH_3_ components with NO_X_ emissions. It also increases the NH_3_ conversion efficiency by reducing the exhaust velocity in the SCR system. However, it is important to choose the optimal geometry as it causes a pressure drop in the exhaust system. Thus, many studies have been analyzed in the literature on the use of static mixers in SCR systems, and the effects of these components on NH_3_ conversion efficiency and pressure drop have been investigated (Park et al. 2014; Tan et al. 2018; Zhu et al. 2020). Prabhu et al. (2017) examined the NH_3_ conversion efficiency parametrically at different exhaust temperatures, spray angles, and injector positions. Parametric studies were compared at 300 °C, 350 °C, and 400 °C exhaust temperatures and 25°, 80°, and 140° spray angles. The highest amount of NH_3_ formation was obtained at 400 °C temperature, 140° spray angle, and injector positions close to the mixing zone. Different catalysts are used for NH_3_/NO_X_ mixture and effective NO_X_ reduction in SCR systems. Devadas et al. (2005) and Wang et al. (2020) investigated the NO_X_ conversion efficiency with Fe-ZSM-5 catalyst, Sultana et al. (2013) with Cu/Fe-ZSM-5 catalyst, Guo et al. (2019) with V_2_O_5_/WO_3_/TiO_2_ catalyst, and Wei et al. (2022) with Mn-based catalyst. Yang et al. (2023) using the Fe_1_–N_4_–C structure and Fe–N_4_–C catalyst/H_2_O_2_ system, created the finest single-atom catalysts (SACs) for NO oxidation, demonstrating the practical and effective usage of H_2_O_2_. Yang et al. (2022) examined single-atom catalysts (SACs) that hold great promise as they exhibit selectivity and efficient atom usage, enabling the transformation of complex molecules with high specificity at a lower expense. In the studies, the NO_X_ reduction performance of the catalyst and the undesirable harmful gases that may occur were investigated comparatively.

Reducing NO_X_ emissions in SCR systems is directly related to NH_3_ conversion. NO_X_ emissions and NH_3_ emissions must react without undesired gases in the catalyst outlet. Therefore, various studies have been proposed to ensure efficient NH_3_ conversion in SCR systems. This conversion is usually made efficient with static mixers and UWS injection parameters. However, to the best of our knowledge, there are studies in the scientific literature on SCR reactor geometry design and the effect of catalyst parameters, but comprehensive studies are necessary. Unlike the studies in the literature, in this study, NH_3_ and NO_X_ conversion efficiency in SCR systems was carried out numerically with SCR design parameters. As a novelty, parametric studies for determining the SCR system diameter, the SCR output form, and mixing zone length and location were examined. Additionally, instead of using different catalysts, the effect of activation energy on NO_X_ conversion efficiency was investigated, and studies were conducted to select possible catalysts to be used in SCR systems. Variable parametric studies were performed for SCR system diameter, SCR output form, and mixing zone dimensions. In addition, the SCR activation energy varies between 7 × 10^3^ and 2 × 10^7^ kJ/kmol. Numerical studies were verified with experimental data from the literature, and then, parametric studies were carried out. Computational fluid dynamics analysis was carried out using the finite volume method with Ansys-Fluent software (ANSYS Inc. 2015).

## Methodology

The SCR system design was carried out for a two-stroke marine diesel engine. The specifications of the diesel engine are given in Table 1. Parametric studies were implemented under 85% load conditions of the diesel engine.

**Table 1:** Specifications of the investigated marine diesel engine

Specification	Unit	Property
Engine type	–	2 Stroke (5G45ME)
Bore	mm	450
Stroke	mm	2250
Power	kW	5400 (at 100% load)
Turbocharger	–	MAN TCR 22-21

**Fig. 1:**
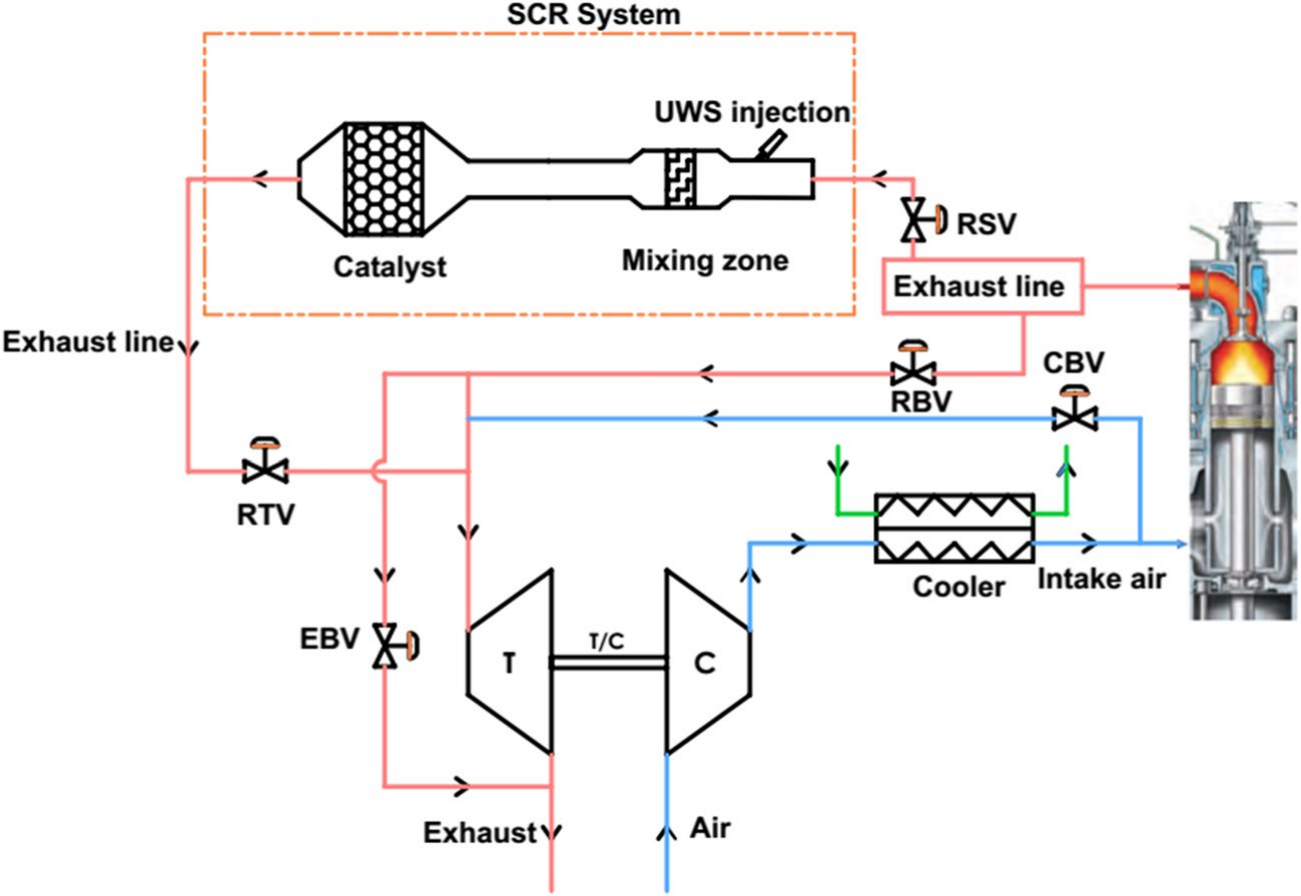
Schematic diagram of HP-SCR

In the HP-SCR system, the SCR is located in the diesel engine exhaust manifold. Thus, the efficiency of UWS evaporation, thermolysis, and hydrolysis reactions increases with high temperature. In the LP-SCR system, on the other hand, SCR is located at the turbocharger outlet, and therefore, UWS decomposition and NO_X_ reactions cannot be carried out effectively with the decrease in temperature. The HP-SCR system is located directly at the low-speed diesel engine exhaust outlet as shown in Fig. 1. Therefore, since the HP-SCR system output will be connected to the turbocharger turbine, the placement of the SCR system is an important factor. Reactor bypass valve (RBV), reactor seal valve (RSV), reactor throttle valve (RTV), cylinder bypass valve (CBV), and exhaust bypass valve (EBV) used in HP-SCR in ship diesel engines are used in system optimization (Bayramoğlu and Özmen 2021).

### Numerical setup

**Fig. 2:**
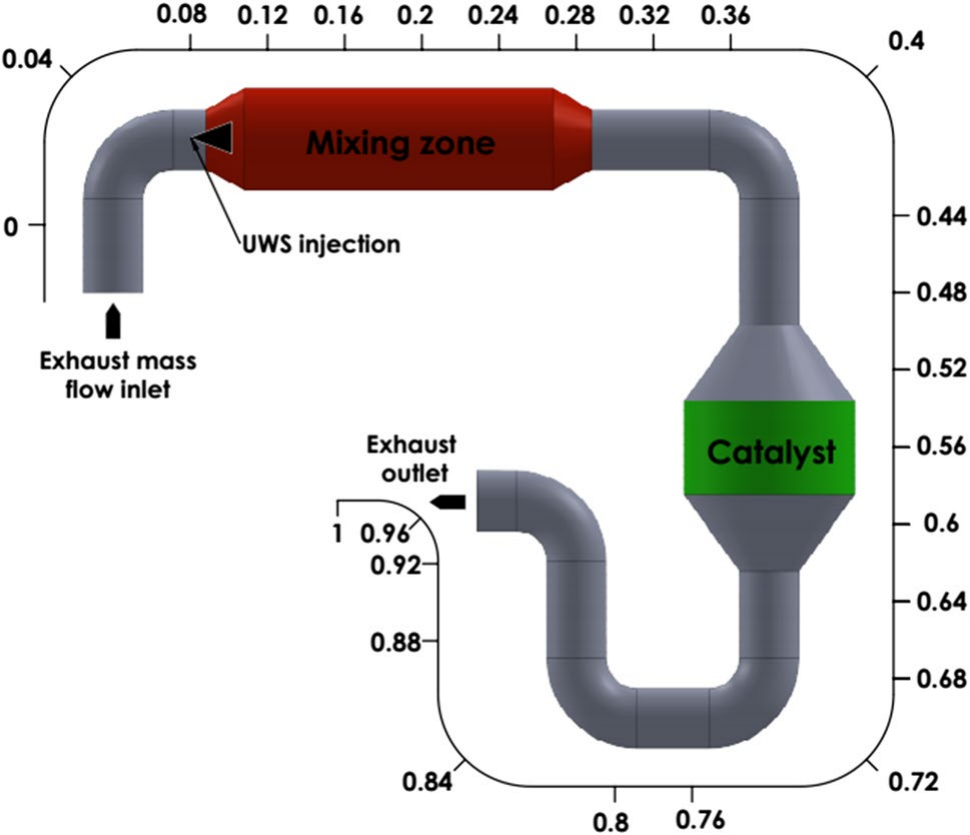
Computational domain of HP-SCR

The SCR system is also designed to include the mixing zone and SCR catalyst. While the mixing zone is the part where the exhaust velocity decreases and UWS decomposition reactions take place effectively, the catalyst is the part where SCR reactions occur. A total of 26 planes were placed on the HP-SCR system, and the data were taken as area-weighted average quantity of the chemicals on each plan. SCR exhaust inlet, exhaust outlet, and UWS injection nozzle locations are also shown in Fig. 2.

UWS consists of 40% urea and 60% water. The process of injecting the UWS exhaust system is provided by the discrete phase model (DPM) (Zahari et al. 2018). In UWS, water evaporates with the exhaust gas temperature. The water in the UWS evaporates with the heat of the exhaust gas. The main parameter affecting UWS evaporation is droplet size. The UWS droplet distribution was based on the Rosin-Rammler particle distribution since it is the most suitable model for determining droplet size and distribution in the literature (Kim et al. 2004). After the evaporation of water, urea turns into NH_3_ and CO_2_ components because of the first thermolysis and then hydrolysis reactions. Equations (1) and (2) show the thermolysis and hydrolysis reactions, respectively (Birkhold et al. 2007).

1 $${\left({{\text{NH}}}_{2}\right)}_{2}{{\text{CO}}}_{\left(\mathrm{s\;or\;l}\right)}\to {{\text{NH}}}_{3\left({\text{g}}\right)}\;+\;{{\text{HNCO}}}_{\left({\text{g}}\right)}$$

2 $${{\text{HNCO}}}_{\left({\text{g}}\right)}\;+\;{{\text{H}}}_{2}{{\text{O}}}_{\left({\text{g}}\right)}\to {{\text{NH}}}_{3\left({\text{g}}\right)}\;+\;{{\text{CO}}}_{2\left({\text{g}}\right)}$$

Catalysts used in the reduction of NO_X_ emissions are generally made of manganese, zinc, or vanadium-based elements in the form of porous media. Two basic reactions occur between NO_X_ and NH_3_ chemicals in SCR reactors. The first of these is the “standard” SCR and fast SCR reaction, which are given in Eqs. (3) and (4), respectively (Olsson et al. 2008).

3 $${4{\text{NH}}}_{3}\;+\;4{\text{NO}}+\;{{\text{O}}}_{2}\;\to\;{4{\text{N}}}_{2}\;+\;6{{\text{H}}}_{2}{\text{O}}$$

4 $${2{\mathrm{NH}}}_{3}\;+\;{\mathrm{NO}}\;+\;{{\text{NO}}}_{2}\to {2{\mathrm{N}}}_{2}\;+\;3{{\text{H}}}_{2}{\text{O}}$$

Thermolysis, hydrolysis, and NO_X_ reactions occur after the evaporation of the UWS injected from the injector. The Arrhenius equation is used in the numerical model to represent these reactions as a function of temperature (McAllister 2011). The Arrhenius expression is given in Eq. (5).

5 $$k={Ae}^{\frac{{-E}_{a}}{{R}_{u}T}}$$

The species transport method is the model that defines the sources of conduction, transport, and chemical reaction for each species in the SCR numerical model with the conservation of mass. The definition of the exhaust gas mixture and chemical species in the SCR system are provided by this model. The reactions between the species in the control volume are described by the volumetric reactions in this model. Reynolds-averaged Navier-Stokes (RANS)-based *k-*ε turbulence model was selected in the analyses. By solving two distinct transport equations, the two-equation *k*-ε turbulence model permits the determination of both the turbulence length and the time scale. The standard *k*-ε turbulence model is a semi-empirical model that has a wide range of applications in terms of providing accurate solutions to heat transfer and industrial flow problems and having advantages in terms of problem-solving time (Ansys, 2015). The numerical analysis study was carried out with the coupling algorithm under steady-state conditions.

**Fig. 3:**
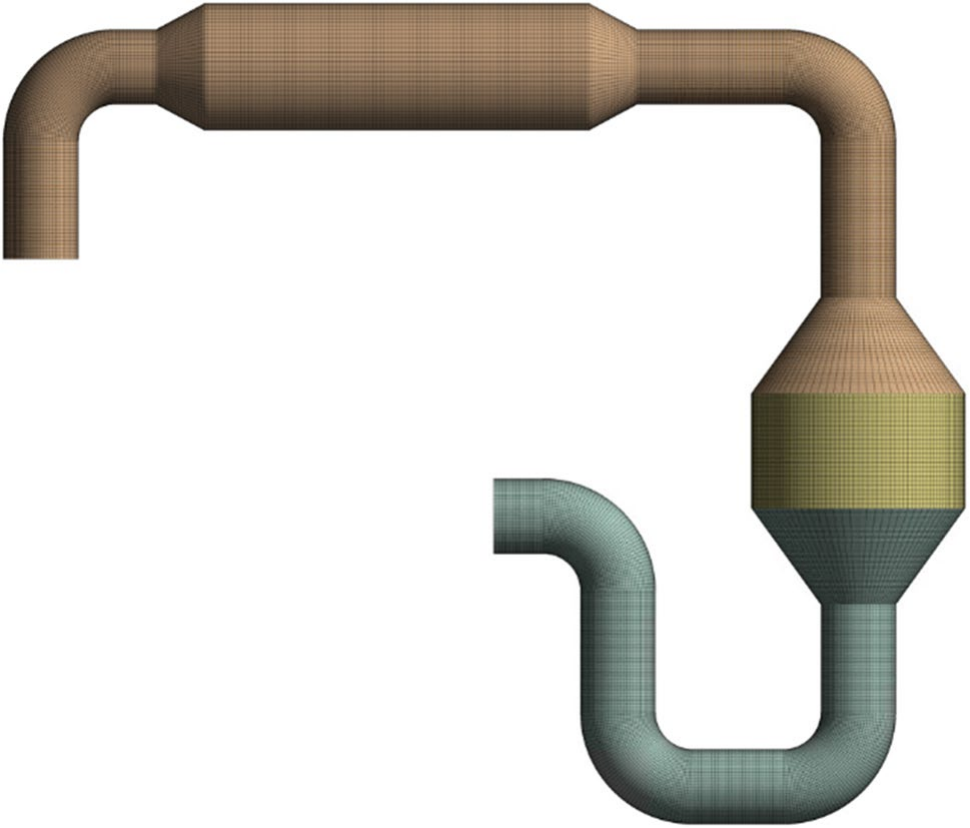
Computational model

The computational model for the HP-SCR system was constructed with structural meshes using the meshing module of the software. The element number is determined as 1M elements, and the mesh structure is given in Fig. 3. The number of elements has been increased to characterize the flow more accurately in the elbow regions. As a method, the model mesh was realized with sweep mesh. The sweep mesh method is used to obtain a homogenous distribution in the mesh.

### Governing equations

For an incompressible, unsteady, two-phase turbulent flow in the SCR system numerical model, the three-dimensional RANS-governing equations for mass, momentum, and energy are solved. The conservation of mass (continuity) equation for a differential element of fluid in the Cartesian coordinates is expressed as Eq. (6).

6 $$\frac{\partial \rho }{\partial t}+\frac{\partial }{{\partial x}_{j}}\left(\rho {u}_{j}\right)=0$$

where $\rho$ is density, $t$ is time, and ${u}_{j}$ is the velocity vector. The momentum equation, also known as the Navier-Stokes equation in flow problems, is given in Eq. (7) with the assumption of incompressible flow.

7 $$\frac{\partial }{\partial t}\left(\rho {u}_{i}\right)+\frac{\partial }{{\partial x}_{j}}\left(\rho {u}_{i}{u}_{j}\right)=\frac{\partial P}{{\partial x}_{i}}+\mu \frac{{\partial }^{2}{u}_{i}}{{\partial x}_{j}{\partial x}_{j}}+\rho {f}_{i}$$

In the given equation, *P* is pressure, ${f}_{i}$ is the source term, and $\mu$ is viscosity. Then, the expression of energy conservation can be given as in Eq. (8).

8 $$\frac{\partial }{\partial t}\left(\rho i\right)+\frac{\partial }{{\partial x}_{j}}\left(\rho i{u}_{j}\right)=-P\frac{\partial {u}_{j}}{{\partial x}_{j}}+\frac{\partial }{{\partial x}_{j}}\left(k\frac{\partial T}{{\partial x}_{j}}\right)+\rho {f}_{i}{v}_{i}+\frac{\partial ({\tau }_{ji}{v}_{i})}{{\partial x}_{j}}$$

where $k$ is the conduction coefficient, $i$ is the internal energy, and $\tau$ is the stress tensor (Baleta et al. 2017). A porous structure is employed to represent the catalyst that was used in the investigation. The momentum expression is given in Eq. (9) for the porous media model (Das et al. 2018).

9 $${S}_{i}=\sum\limits_{j=1}^{3}{D}_{ij}\mu {u}_{j}+\sum\limits_{j=1}^{3}{C}_{ij}\frac{1}{2}\rho {u}_{j}{u}_{j}$$

Equation (9) is rearranged and written as Eq. (10) according to Darcy’s law. The relation between velocity and pressure drop is expressed by this law in the SCR system.

10 $$\Delta p=-\left(\frac{\mu }{a}v+{C}_{2}\frac{1}{2}\rho {v}^{2}\right)\Delta m$$

### Boundary conditions

The boundary and initial conditions used in the numerical study are given in Table 2. The given boundary conditions are classified to include inlet, outlet, injecting parameters, and porous media properties. Inlet temperature and mass flow rate are given for 85% load conditions. Experimental exhaust data were obtained from MAN energy solutions.

**Table 2:** Boundary and initial conditions

Boundary and initial conditions			Unit	Condition and value
Inlet	Mass flow inlet		kg/s	10.4
	Temperature		K	660
	Gas compositions	NO_X_	ppm	1500
		SO_X_		600
		O_2_	%	13
		CO_2_		5.2
		H_2_O		5.35
Outlet			–	Pressure outlet
Wall			–	Adiabatic wall
Catalyst	Pressure-jump coefficient (C_2_)		1/m^2^	1.12E+07
	Permeability (1/α)		mm	3.72
Injection	Composition		–	60% water-40% urea
	Rate		l/h	90
	Mean diameter		mm	0.022
	Spread parameter		–	3.27
	Velocity		m/s	10.6
	Cone angle		°	70

The outlet was accepted as the pressure outlet, and the pressure drop was calculated regarding this boundary condition. Exhaust gas components consist of the dominant gases formed because of the basic combustion process and the NO_X_ component that is required to be reduced. Since NO is the dominant species in NO_X_ emissions, NO_X_ has been accepted as 90% NO and 10% NO_2_ (Zhu et al. 2019). The wall boundary condition is assumed in SCR systems as an adiabatic wall. The catalyst is defined as porous media, Besides, porous media boundary conditions due to pressure drop are specified. The injection parameters which are given in the Table 3 were verified with the experimental data given in the model validation section. Parameters *A* and *E* for the UWS decomposition and SCR reactions which are expressed in Eqs. (1)–(4) are given in Table 3 (Yim et al. 2004).

**Table 3:** Reaction parameters of UWS decomposition and SCR

Reaction	Thermal		Catalytic	
	${A}_{i}$	${E}_{i} [{\text{j}}/{\text{kmol}}$]	${A}_{i}$	${E}_{i} [{\text{j}}/{\text{kmol}}$]
Thermolysis	4.9E+03	2.3E+07	4.5E+03	2.26E+07
Hydrolysis	2.5E+05	6.22E+03	3.1E+04	1.58E+07
Standard SCR	–	–	2.3E+08	8.49E+04
Fast SCR	–	–	1.9E+12	8.51E+04

## Results and discussion

### Validation of the model and mesh independence study

**Fig. 4:**
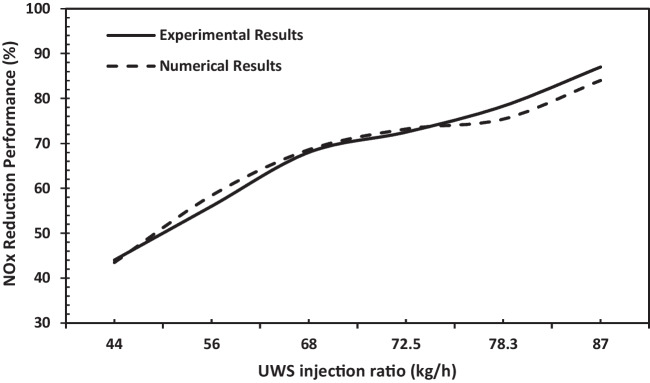
Validation of numerical model

The validation study of the numerical model, whose parameters are given in the boundary conditions section, has been accomplished with the experimental study from the literature (Sung et al. 2020). The numerical study was applied under six different UWS injection ratios, the same as the experimental study. In the numerical study, the NO_X_ reduction performance was calculated using the parameters given in the boundary conditions section. It was determined that the results of the numerical and experimental studies were compatible with each other, and the results are given in Fig. 4. One can infer from the results that the maximum error is 3.4% with 87 l/h of the UWS injection ratio.

**Fig. 5:**
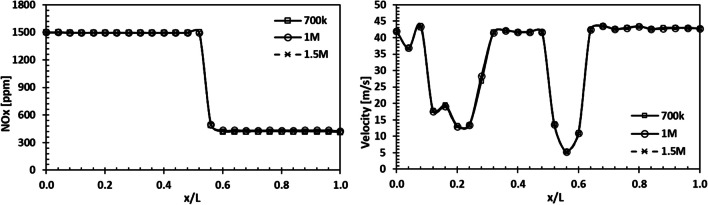
Variation of NO_X_ and velocity with the number of mesh elements

Mesh independence analysis was performed for the HP-SCR system in 700k, 1M, and 1.5M element numbers. Mesh independency results for variation of NO_X_ emissions and velocity along the SCR system are demonstrated in Fig. 5. It is shown that the results of the simulation agree well with the experimental data, and 1M element number was used for parametric studies.

### Determination of SCR system diameters

**Fig. 6:**
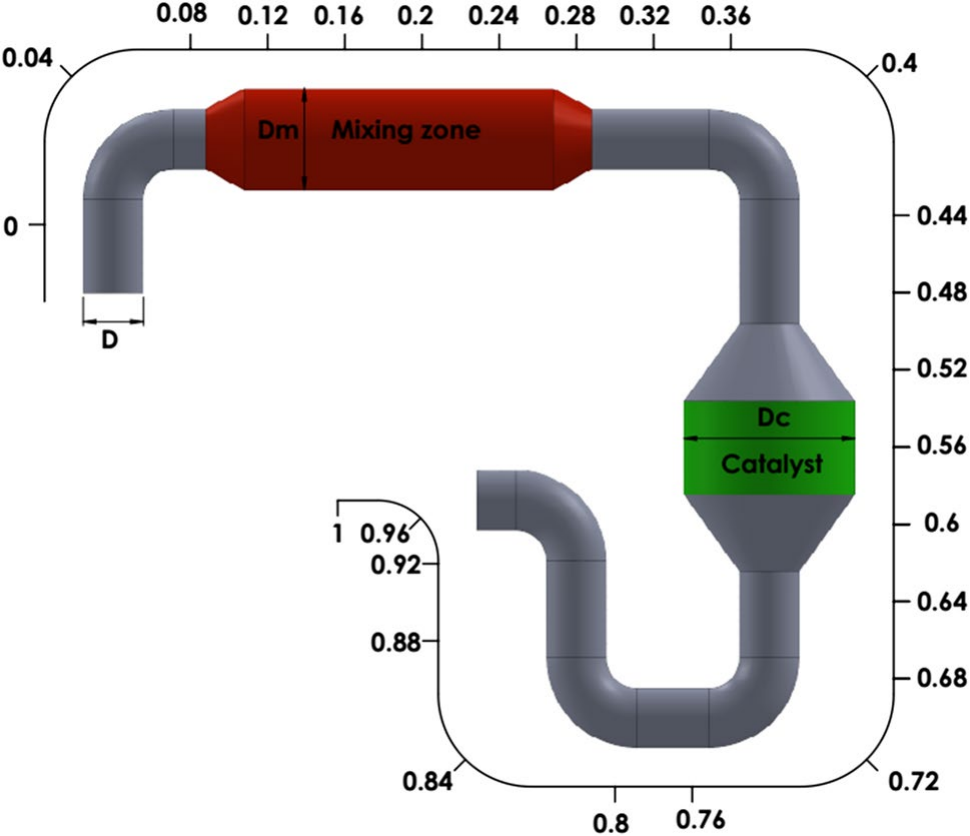
SCR system and locations of investigated variables

Determining the flow rate in pipes plays a critical role in determining the flow characteristics such as velocity and friction on the walls. In this section, parametric studies were performed on variable system diameters to determine the flow velocity and flow characteristics in the SCR system. Parametrically investigated variables such as *D*, *D*_*m*_, and *D*_*C*_ are given in Fig. 6 for the SCR system.

In the parametric studies, the effect of five different parameters on the system performance was investigated. Here, *D* is the system diameter, *D*_*m*_ is the mixing zone diameter, and *D*_*C*_ is the SCR diameter. Dimensions of five different parameters are given in Table 4 for the SCR system.

**Table 4:** Dimensions of the five different parameters

No.	System diameter (*D*) (mm)	Mixing zone diameter (*D*_*m*_) (mm)	SCR diameter (*D*_*C*_) (mm)
1	540	900	1540
2	620	1050	1770
3	700	1190	2000
4	780	1320	2220
5	860	1460	2450

**Fig. 7:**
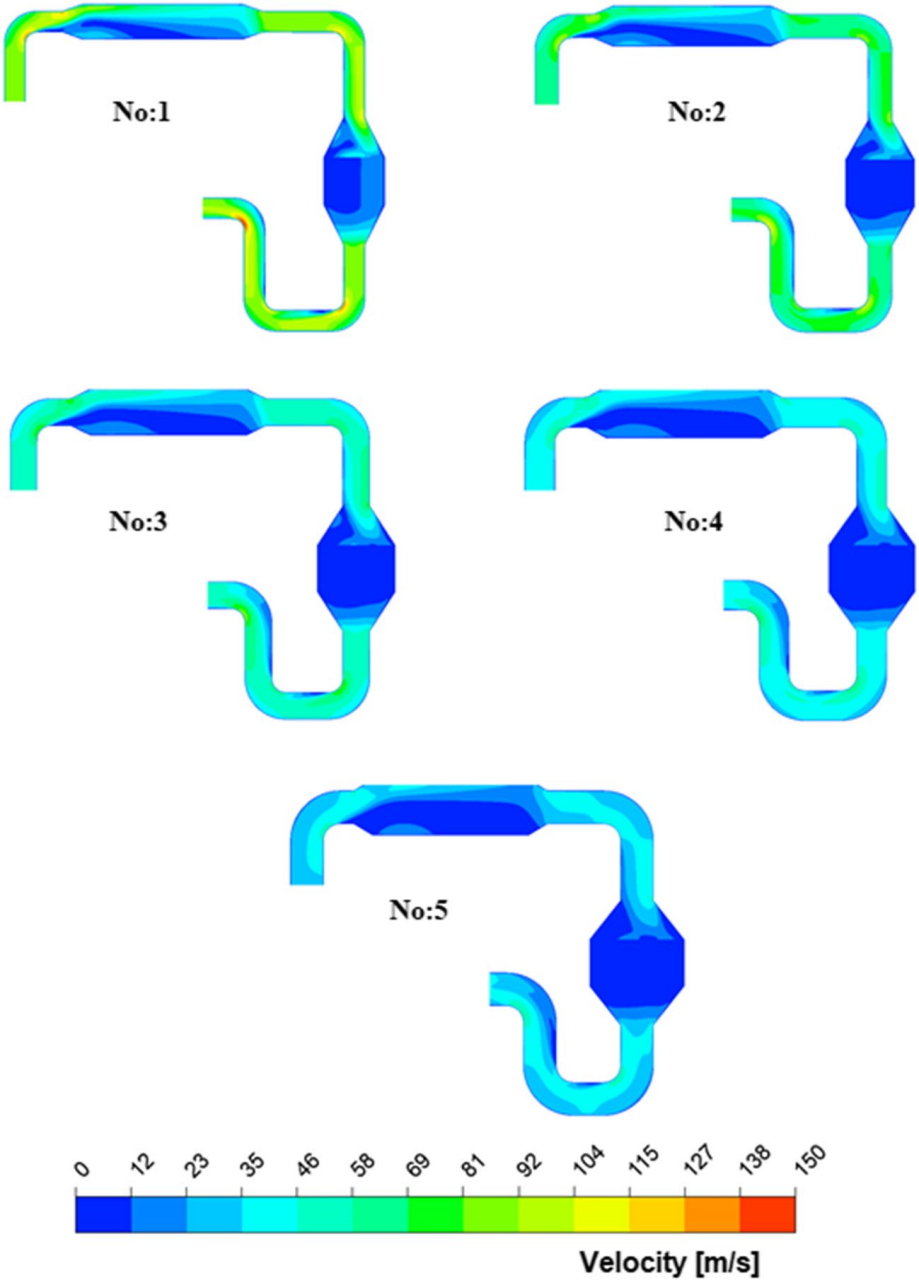
Distribution of velocity profile for variable diameters

The diameters that are shown in Table 4 are proportional to each other, and the effect of each diameter condition on the NO_X_ reduction performance and loss of pressure was examined. Additionally, velocity profiles along with the geometries at each diameter value were also examined, and their variation throughout the system was determined. Figure 7 shows the velocity distribution for variable diameters throughout the SCR system.

**Fig. 8:**
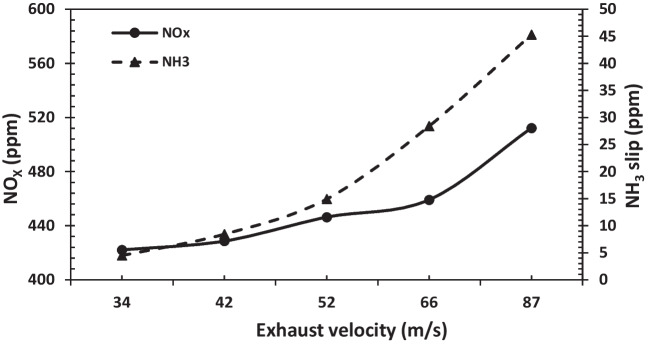
NO_X_ and NH_3_ slip versus exhaust velocity

When the velocity distribution along the system was identified, it was clearly seen that the velocity decreased at the entrance of the mixing zone and reached its minimum value after the catalyst. The decrease in the flow rate in the mixing zone allows the reactions to take place more effectively. From the velocity profiles obtained, it is clearly seen that the flow velocity varies inversely with the system diameter. Moreover, the inhomogeneity of the velocity distribution at the catalyst inlet is due to the elbow geometry before the catalyst. While the average velocity value throughout the system was approximately 87 m/s for system no. 1, it was determined to be 34 m/s for system no. 5. The amounts of NO_X_ and NH_3_ slip depending on exhaust velocity are given in Fig. 8 for the SCR system.

**Fig. 9:**
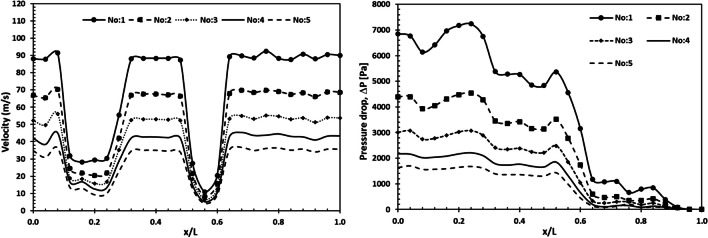
Change of pressure drop and velocity throughout the SCR system

Higher exhaust velocity leads to increased NO_X_ emissions and NH_3_ slip. The increasing diameter of the SCR system reduces the exhaust velocity and allows the UWS decomposition reactions to occur effectively in the SCR system. It became clear that NO_X_ emissions and the change in NH_3_ slip were low up to a speed of about 42 m/s, while the NO_X_ and NH_3_ slip increased at a velocity higher than 42 m/s. The results show that the SCR system diameter of 780 mm is the most optimal value. Figure 9 shows the pressure drop and velocity for each diameter value throughout the SCR system. In the SCR system, the pressure drop decreases as the diameter increases. Therefore, increasing the system diameter reduces the pressure drop, which directly affects engine performance. However, it is undesirable to increase the diameter value in terms of HP-SCR system location.

### Effect of catalyst outlet form on NO_X_ emissions and pressure drop

**Fig. 10:**
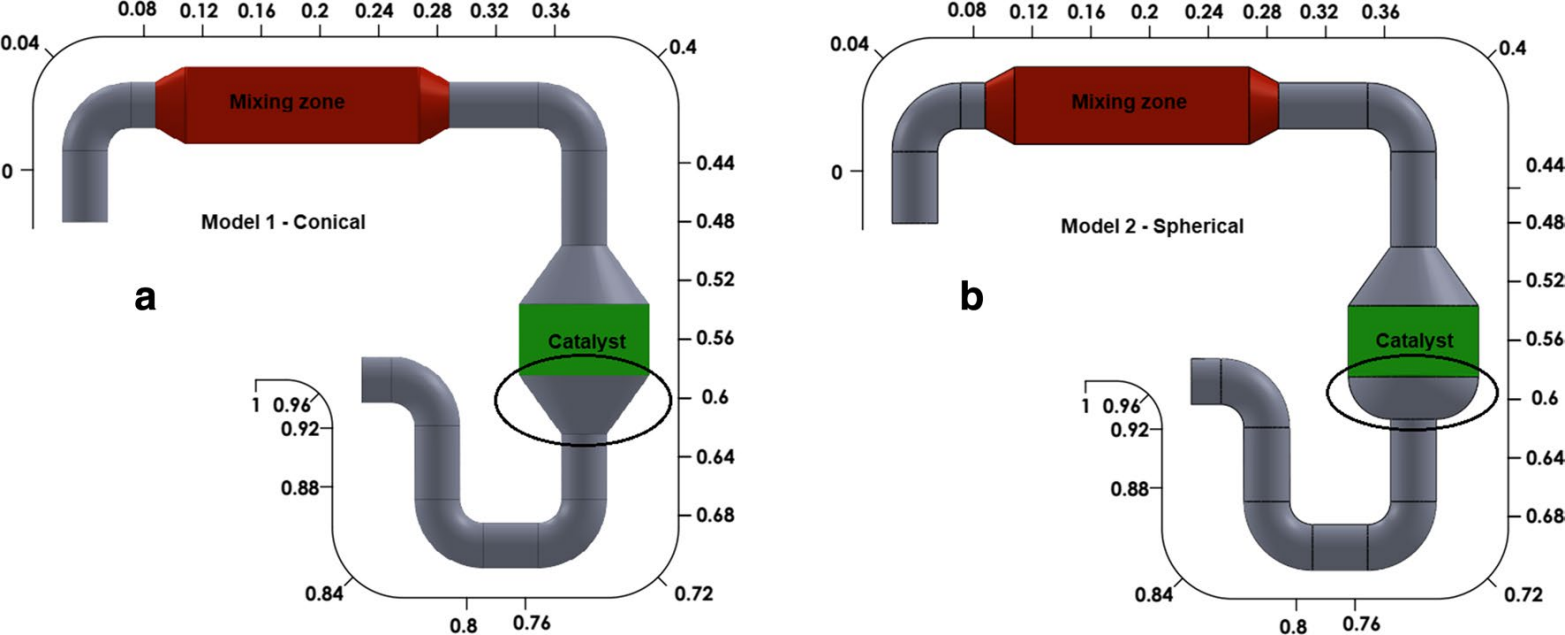
Variable catalyst outlet forms **a** model 1 and **b** model 2

To examine the effect of the catalyst outlet form on the SCR system performance, two different geometries indicated in Fig. 10 were compared. Selected catalyst geometries are two different forms frequently used in SCR catalysts. System parameters were taken as constant for both catalyst geometries.

**Fig. 11:**
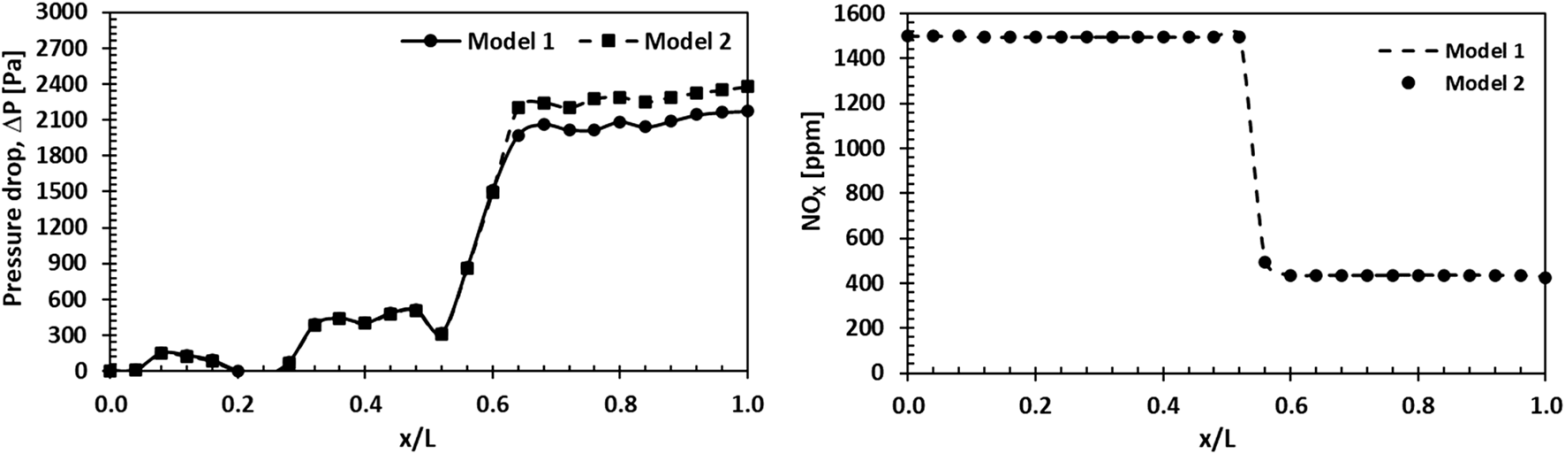
Deviation of the pressure drop and NO_X_ emissions throughout the SCR system

Figure 11 shows the deviation of NO_X_ emission and pressure drop through the SCR system for two different geometries. NO_X_ emissions are nearly at the same level in both cases. Furthermore, the results indicate that the SCR reactions are completed in the SCR catalyst. Although NO_X_ emissions are the same for both geometries, there are differences between the pressure drops. For model 2, where the catalyst outlet geometry is spherical, the pressure drop is higher than for model 1. Therefore, the conical catalyst outlet geometry represents the most suitable geometry between the two systems.

### Effect of catalyst activation energy on NO_X_ reduction performance

**Fig. 12:**
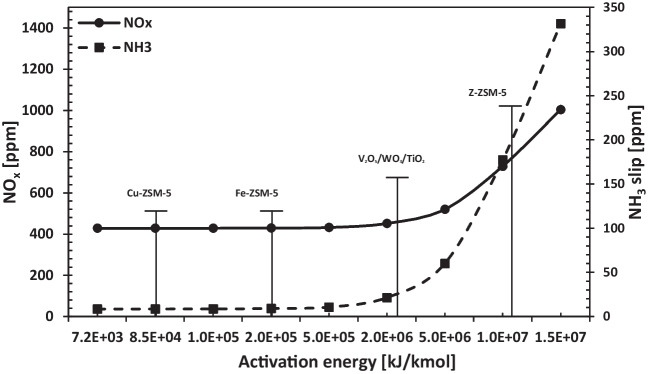
Change of NO_X_ and NH_3_ slip versus catalyst activation energy

Catalysts used in SCR systems provide the formation of free N_2_ and H_2_O by reacting NO_X_ and NH_3_ components. However, for NO_X_ and NH_3_ components to react without using any catalyst in the system, the required exhaust temperature must be between 875 and 1050 °C. NO_X_ reduction systems operating in these temperature ranges are named selective non-catalytic reduction (SNCR) systems, and SNCR systems are used only in large industrial plants such as furnaces and boilers (Hao et al. 2015). The NO_X_ reduction values and NH_3_ shift of the system catalyst operating under high-pressure conditions of a two-stroke diesel engine for different activation energies are given in Fig. 12. The findings show that catalysts with 5 × 10^6^ kJ/kmol and lower activation energy increase both NO_X_ and NH_3_ slip.

**Fig. 13:**
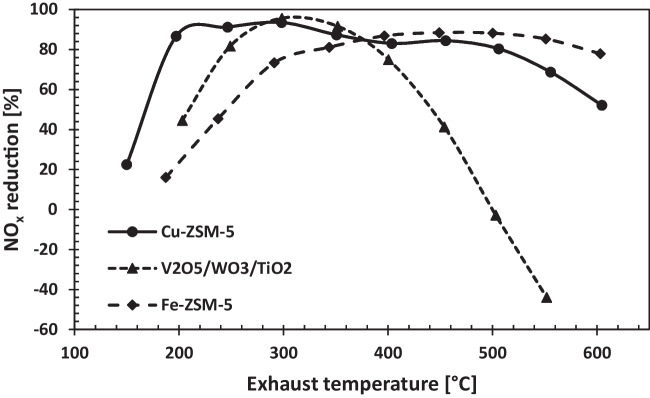
NO_X_ reduction performance of variable catalyst

When the literature is examined, the most preferred catalyst types in SCR systems are Cu-zeolite, Fe-zeolite, H-zeolite, and V_2_O_5_/WO_3_/TiO_2_. Various catalysts are given on the graph according to the reduction ratios and activation energies. H-ZSM-5 catalyst reduction data was taken from a study by Jabłońska et al. (2021), the comparison of Fe-ZSM-5 and Cu-ZSM-5 catalysts was obtained from Sultana et al. (2013), the activation energy for the Fe-ZSM-5 catalyst was taken from the study by Bulushev et al. (2002), and finally, the V_2_O_5_/WO_3_/TiO_2_ data were taken from the study by Kamasamudram et al. (2010). In addition, the NO_X_ reduction performance of the Cu-ZSM-5 catalyst used in the study, depending on the temperature, is demonstrated in Fig. 13.

The obtained data show that the NO_X_ reduction performance of the Cu-ZSM-5 catalyst is better than other catalysts in wide temperature ranges. Therefore, Cu-ZSM-5-type catalyst is the most suitable catalyst to use according to the data obtained for SCR systems with exhaust gas temperature in the temperature range of 300–400 °C. Moreover, another point that should be considered is that different metal mixtures and more suitable catalysts with different activation energies can be developed for SCR systems and can be used to reduce NO_X_. The Cu-ZSM-5 catalyst, which is commonly employed as an SCR catalyst in the literature, functions across a wide temperature range. The primary function of catalysts is to ensure that SCR reactions proceed efficiently by lowering the activation energy at low temperatures. In this context, the study indicated that Cu-ZSM-5 catalysts running at around 250 °C exhaust temperature were appropriate for this system. Furthermore, the Cu-ZSM-5 catalyst is the most often employed due to its low-temperature denitration capability (Yuanyuan et al. 2024). Additionally, the usage of this catalyst in increasing exhaust temperatures and varied engine types is seen as significant.

### Effect of mixing zone position and length on system performance

**Fig. 14:**
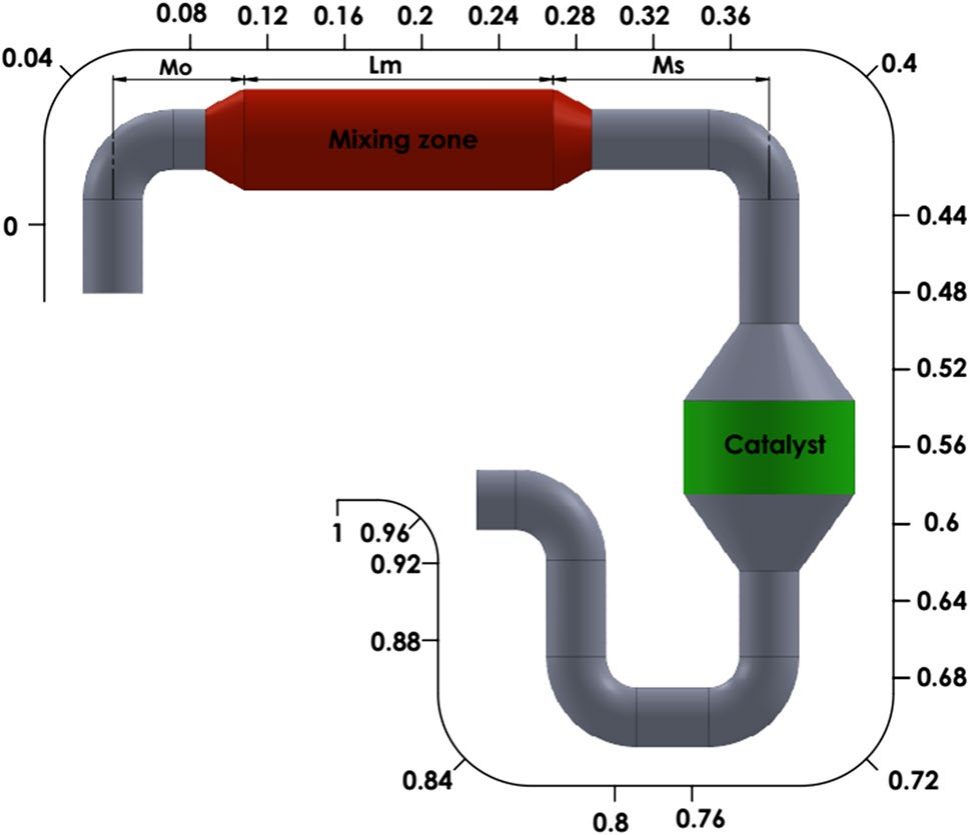
Dimensions of mixing zone length and locations

Five different parametric studies were conducted to examine the effects of variable mixing zone length and location on thermolysis and hydrolysis reactions. Figure 14 represents the mixing zone length and locations. Variable dimensions for each condition are given in Table 5.

**Table 5:** Variable dimensions of mixing zone

No.	Units	*M*_*O*_	*L*_*m*_	*M*_*S*_
1	mm	1200	4000	2300
2	mm	1500	4000	2000
3	mm	1500	3400	2600
4	mm	1800	2800	2900
5	–	No mixing zone		

**Fig. 15:**
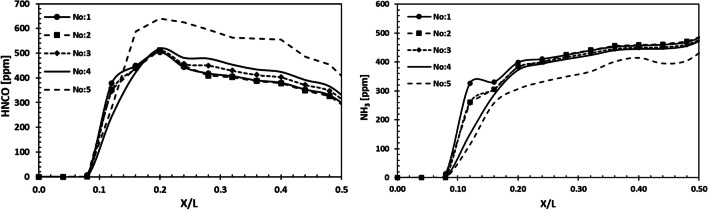
Variation of HNCO and NH_3_ throughout the SCR system

The variation of HNCO and NH_3_ components in the SCR system is presented utilizing the analyses made with variable parametric measures. Figure 15 shows the variation of HNCO components formed because of the thermolysis reaction for the length from the SCR inlet to the catalyst inlet. It was observed that the highest HNCO formation was for the geometry without a mixing zone. The decrease in mixing zone length indicates that the amount of HNCO increases throughout the system.

The condition with the least amount of HNCO is valid for the design conditions where the catalyst length is 4000 mm. HNCO chemicals formed because of thermolysis reactions in SCR systems are then converted into NH_3_ components. Therefore, the HNCO components should decrease at the catalyst entrance and convert to NH_3_. In the same way, system performance depends on the NH3 conversion efficiency. The higher the NH_3_ formation at the catalyst inlet, the better the NO_X_ reduction performance. Therefore, with low catalyst lengths, the amount of NH_3_ conversion is lower than other ratios. The large mixing zone size also increases the amount of NH_3_ components.

## Conclusion

This study proposes a different perspective to investigate the effect of system design parameters on NH_3_ conversion performance. The effects of different SCR system diameters, mixing zone location and size, and variable catalyst activation energies on SCR performance parameters were investigated numerically. Variable dimensions are used in SCR systems to provide NH3 and NO_x_ conversion. The most important component that ensures this conversion is static mixers. These mixers provide NH3 conversion by creating turbulence. The findings of this investigation are given below:

- In the SCR system, the effects of the SCR system diameter variation and the flow velocity on the system performance were investigated. As the pipe diameter increased, NO_X_ emissions and pressure drop decreased. The most suitable system diameter was determined as 780 mm.
- While the catalyst outlet form did not affect the reduction of NO_X_ emissions much, a higher pressure drop occurred in the spherical outlet form compared to the conical outlet form.
- The main function of the catalysts used in SCR systems is to reduce the NO_X_ emissions in the exhaust gas by lowering the threshold energy of the reactions that lead to the formation of NO_X_ and NH_3_ components. In parametric studies performed for variable catalyst activation energy, it was determined that catalyst structures with 5·10^6^ kJ/kmol and below activation energies are suitable models for SCR systems operating under high-pressure conditions.
- NO_X_ reduction performances at different exhaust temperature ranges were compared for three different catalyst types taken from the literature. Thus, it was determined that the Cu-ZSM-5 catalyst was the most suitable catalyst for the given exhaust temperatures of the diesel engine.
- Finally, the effects of various mixing zone lengths and locations on the system UWS decomposition were investigated. In the parametric studies, five different positions and lengths were taken, and the variation of two crucial species such as HNCO and NH_3_, which are formed with thermolysis and hydrolysis reactions throughout the system, were investigated. The results show that the system parameters where the *M*_*O*_, *L*_*m*_, and *M*_*S*_ dimensions are 1200, 4000, and 2300 mm, respectively, provide the most efficient NH_3_ conversion efficiency.

Determining which specific sites on the catalyst surface are responsible for catalyzing the reaction, temperature, pressure, and reactant concentration influence is crucial. It becomes challenging to optimize catalyst design for desired reaction outcomes, such as increased selectivity, efficiency, and stability. Future studies of SCR systems on different types of ships and on ship engines using alternative fuels could be realized. Advancing studies on catalyst development and reaction mechanisms can indeed contribute to achieving UN Sustainable Development Goal 13 (Climate Action) in the maritime sector in several ways.

## Data Availability

Data that support the findings of this study will be made available from the Authors on reasonable request.
